# Functional annotation of breast cancer risk loci: current progress and future directions

**DOI:** 10.1038/s41416-021-01612-6

**Published:** 2021-11-05

**Authors:** Shirleny Romualdo Cardoso, Andrea Gillespie, Syed Haider, Olivia Fletcher

**Affiliations:** The Breast Cancer Now Toby Robins Research Centre, The Institute of Cancer Research, London, UK

**Keywords:** Breast cancer, Genome-wide association studies

## Abstract

Genome-wide association studies coupled with large-scale replication and fine-scale mapping studies have identified more than 150 genomic regions that are associated with breast cancer risk. Here, we review efforts to translate these findings into a greater understanding of disease mechanism. Our review comes in the context of a recently published fine-scale mapping analysis of these regions, which reported 352 independent signals and a total of 13,367 credible causal variants. The vast majority of credible causal variants map to noncoding DNA, implicating regulation of gene expression as the mechanism by which functional variants influence risk. Accordingly, we review methods for defining candidate-regulatory sequences, methods for identifying putative target genes and methods for linking candidate-regulatory sequences to putative target genes. We provide a summary of available data resources and identify gaps in these resources. We conclude that while much work has been done, there is still much to do. There are, however, grounds for optimism; combining statistical data from fine-scale mapping with functional data that are more representative of the normal “at risk” breast, generated using new technologies, should lead to a greater understanding of the mechanisms that influence an individual woman’s risk of breast cancer.

## Background

Genome-wide association studies (GWAS, Table 1) coupled with large-scale replication and fine-scale mapping studies (Table 1) have led to the identification of more than 150 genomic regions that are associated with breast cancer risk [1–6]. Efforts to translate these findings to improve the lives of women who are at risk of developing breast cancer are focused on two main areas: risk prediction and a greater understanding of disease mechanism. The subject of this review is the latter.

**Table 1:** Definitions.

SNP	Single-nucleotide polymorphism: variation at a single nucleotide in the DNA sequence; differs between individuals within a population. By definition, a polymorphism occurs at a frequency greater than 1% in the population.
Germline variation	Variants that are inherited from the parents and by definition, therefore, present in a reproductive cell (ovum or sperm) in one parent.
Somatic mutation	A variant that occurs de novo in somatic cells of an individual (all cells of the body except the gametes).
Copy-number variation	A type of structural variation; specifically, a duplication or deletion event that affects a considerable number of base pairs.
Cancer genes	Genes which, based on sequencing of matched “normal” (usually from blood) and tumour DNA, confer a growth advantage to the cancer cells due to somatic and/or germline mutations.
Linkage disequilibrium (LD)	The non-random association of alleles at different loci in a population; i.e., the correlation structure between individual variants that map proximal to each other and are, therefore, co-inherited. Linkage disequilibrium is population-specific.
GWAS	Genome-wide association study: a population-scale study in which variants that are specifically selected to capture to common variation across the genome (through linkage disequilibrium), are genotyped in individuals with and without a phenotype of interest.
Fine-scale mapping	Fine-scale mapping refers to the process by which a GWAS association signal is refined. Specifically, at a given region, a dense panel of variants are selected to be genotyped or imputed and tested for association with outcome.
Credible causal variants (CCVs)	Originally defined in Udler et al. [30] and subsequently used in Fachal et al. [5]; a group of variants that cannot be excluded as “functional” on statistical grounds alone. In practical terms, all variants for which the association *P* value is within two orders of magnitude of the “top SNP” at a given signal.
Functional variant	A variant for which there is evidence (statistical and/or biological) of a causal association (rather than a correlative association, below) with outcome.
Correlated variant	A variant which is associated with outcome through correlation (by linkage disequilibrium) with a “functional” variant.
eQTL	Expression quantitative trait loci: genomic loci which harbour a variant/variants that show an association between genotype (AA/Aa/aa) and levels of expression of a gene (usually quantified as steady-state mRNA levels).
Intermediate phenotype	A quantitative biological trait reflecting the pathway to disease development. Sometimes used as a statistically efficient alternative to a disease outcome.
Cis-association	In the context of an eQTL, a cis-association is an association between genotype and levels of expression of a gene that maps proximal to the genetic variant.
Trans-association	In the context of an eQTL, a trans-association is an association between genotype and levels of expression of a gene that maps distal to, or on a different chromosome from, the genetic variant.
3’ and 5’ UTR	Untranslated regions: UTRs map upstream of the first codon for translation (5’ UTR) and downstream of the last codon for translation (3’ UTR). The 5’ UTR is important for regulating transcription and the 3’ UTR is important for post-translational regulation of the gene.
Promoter	A DNA sequence that binds proteins (including RNA polymerase) that are required to initiate transcription; usually located at the 5’ end of the gene just upstream of the transcription start site.
Transcription start site (TSS)	The location at the 5’-end of a gene sequence at which transcription begins.
Splice donor and acceptor sites	Recognition sites for mRNA processing; donor-splice is the splicing site at the beginning of an intron (5’ end) and acceptor splice is the splicing site at the end of an intron (3’ end).
Enhancer	Regulatory DNA sequence that, when bound by transcription factors, increases gene transcription. Can act in an orientation independent manner (ie an enhancer can be located upstream or downstream of the TSS) and can act over large distances (up to 1 Mb or possibly more).
Transcription factor (TF)	Sequence-specific DNA-binding proteins that regulate transcription of a gene by binding to enhancers or promoters.
eRNA	Enhancer-derived RNAs: non-coding RNA transcripts originating from genomic regions that carry active histone modifications (H3K27ac, H3K4me1, H3K4me3) indicative of an active enhancer element. eRNAs can be unidirectional or bidirectional.
Epigenetics	The study of changes in phenotypes caused by modification of gene expression rather than alteration of the genetic code itself.
Promoter hypermethylation	DNA methylation is an epigenetic modification of DNA in which methyl groups are added to the DNA. Methylation can change the activity of a gene without changing the sequence, in particular hypermethylation of CpG islands that map 5’ to a gene promoter is associated with gene silencing.
Episomal	Autonomously replicating extrachromosomal DNA; in the context of the methods described in this review, the important point is that the DNA is not integrated into the genome.
Pluripotent stem cell	Cells that can self-renew and differentiate into any cell in the body.
Cell autonomous	Acting only within the cell in which the gene is expressed, as opposed to influencing the behaviour of surrounding cells.
Exome sequencing	Genomic sequencing of the exons in a genome.
3C	Chromosome-conformation capture: a technique for analysing the spatial organisation of chromatin in the nucleus. 3C is a “one-by-one” technique testing for an excess of interactions between two pre-defined regions of interest.
Hi-C	Genome-wide version of 3C; the “all-by-all” technique for quantifying all possible pairs of interactions across the genome.
DNase-seq	A technique for identifying regions of open chromatin on the basis that nucleosome-depleted DNA at active regulatory regions (promoters and enhancers) is more sensitive to cleavage by DNase I, creating regions of DNase-I hypersensitivity.
FAIRE-seq	Formaldehyde-assisted isolation of regulatory elements: a technique for identifying regions of open chromatin on the basis that formaldehyde cross-linking is less efficient in active nucleosome-depleted DNA than in nucleosome-bound DNA.
ATAC-seq	Assay for transposase-accessible chromatin: a technique for identifying regions of open chromatin on the basis that a hyperactive transposase (Tn5) preferentially cleaves and tags (tagments) regions of open chromatin.
Active histone modifications	Histones can be post translationally modified by methylation, phosphorylation, acetylation, ubiquitylation or sumoylation. Histone modifications are correlated with specific states of activity; acetylation of K27 and mono-methylation of K4 on histone H3 (H3K27ac and H3K4me1) are active enhancer marks, and tri-methylation of K4 on histone H3 (H3K4me3) is an active promoter mark.
CTCF	CCCTC-binding factor: a DNA-binding protein that performs a structural role in genome organisation. Depending on the context, CTCF can also recruit histone acetyltransferase-containing complexes or histone deacetylase-containing complexes and function as a transcriptional activator or repressor, respectively.
ESR1	Oestrogen receptor 1: an oestrogen receptor and ligand-activated transcription factor. One of the transcription factors that define the transcriptome in oestrogen-receptor-positive breast cancer cells.
FOXA1	Forkhead box A1: a pioneer factor that can directly bind condensed chromatin and recruit transcription factors (including ESR1 and GATA3) and histone-modification enzymes. One of three transcription factors that define the transcriptome in oestrogen-receptor-positive breast cancer cells.
GATA3	GATA binding protein 3: a transcription factor originally identified in the regulation of T-cell development. One of three transcription factors that defines the transcriptome in oestrogen-receptor-positive breast cancer cells.

Translating GWAS findings into a greater understanding of the mechanisms that influence an individual’s risk of breast cancer, requires the identification of functional variants (as opposed to correlated variants, Table 1) and the targets of these functional variants (the genes or non-coding RNAs that mediate the associations observed in GWAS). The output from a GWAS is an association signal between a marker single-nucleotide polymorphism (SNP, Table 1) and outcome; in short, a GWAS provides evidence that a particular region of the genome is associated with breast cancer risk but little or no information regarding the mechanism behind this association. The vast majority of GWAS signals map to non-protein-coding regions and are thought to influence transcriptional regulation [7, 8]. With a few exceptions (e.g., splice donor and acceptor sites, Table 1), our ability to predict the likely impact of non-coding variation is extremely limited. To compound this difficulty, marker SNPs are specifically selected to capture common variation at a given region of the genome, and by definition, therefore, will be highly correlated with many other variants, any of which might have a functional association with breast cancer risk. Identifying “targets”, that is, the genes or non-coding RNAs that mediate the associations observed in GWAS, also provides challenges. Regulatory elements can influence the expression of multiple genes, they can “skip over” nearby genes and can act over distances of at least 1 Mb [9, 10]. Indeed, many breast cancer GWAS signals map to gene deserts with the nearest-known protein-coding genes mapping several hundred kilobases (kb) away.

The Breast Cancer Association Consortium (BCAC, http://bcac.ccge.medschl.cam.ac.uk/) was set up as a multidisciplinary consortium of investigators, who are interested in investigating the inherited risk of breast cancer. Led by scientists at the University of Cambridge, BCAC published the first breast cancer GWAS [11] and have led a series of locus-specific follow-up studies [12–29]. Most recently, BCAC carried out a fine-scale mapping analysis of 152 breast cancer risk regions in 109,900 breast cancer cases and 88,937 controls of European ancestry [5]. Using multivariate logistic regression, they confirmed associations for 150 of the 152 regions (*P* < 1 × 10^−4^); to define independent signals within each region, they used stepwise multinomial logistic regression, deriving the association of each variant, conditional on the more significant ones, in order of statistical significance. This analysis reported 352 independent signals, 196 for which the evidence for association was strong (conditional *P* < 10^−6^) and 156 for which the evidence was more modest (10^−6^ < conditional *P* < 10^−4^), with a range of one (*n* = 70) to nine (*n* = 2) independent signals per region. Finally, they defined credible causal variants (CCVs, Table 1) within each signal as variants with conditional *P* values within two orders of magnitude of the index variant [30]. The number of CCVs per signal ranged from one (*n* = 66) to 375, resulting in a total of 13,367 CCVs for follow-up analysis. Overall, these data provide some insight into the scale of the task required if we are to identify the truly functional variants from amongst the plethora of correlated variants, link these variants to their target genes and understand how the temporal, cell-type-specific response stimulated expression of these genes that influence breast cancer risk. Over the past 10 years, however, tools for the annotation of CCVs and the selection of target genes have been developed and publicly funded resources, such as the Encyclopedia of DNA Elements [31] (ENCODE, https://www.encodeproject.org/ Table 2) and Roadmap Epigenomics project (http://www.roadmapepigenomics.org/; Table 2), have coordinated efforts to generate genome-wide datasets for a wide range of the assay and cell types and made these rapidly publicly available, in an effort to facilitate the interpretation of GWAS signals.

**Table 2:** Resources.

ENCODE	The Encyclopedia of DNA Elements (ENCODE) Consortium maintains a portal of publicly available epigenetic datasets from a wide range of assays for identification of functional and regulatory elements, including many variations of RNA-seq, ChIP-seq, DNase-seq and DNA methylation arrays. https://www.encodeproject.org/
Roadmap Epigenomics	The NIH Roadmap Epigenomics Mapping Consortium is a resource that comprises publicly available epigenomic data from primary cells generated using a number of methods, such as histone modification ChIP-seq, RNA-seq and DNA methylation assays. http://www.roadmapepigenomics.org/
Viestra.org	Digital genomic footprinting providing a high-resolution genome-wide consensus transcription-factor footprint index in 243 human cell and tissue types. Accessible through the ENCODE portal and UCSC browser. https://www.vierstra.org/resources/dgf
Descartes	Single-cell ATAC-seq and gene expression data generated in a broad range of human foetal tissues (53 samples representing 15 organs), to create an atlas of linked cell-type-specific enhancers and genes. https://descartes.brotmanbaty.org/bbi/human-chromatin-during-development/
IHEC	The International Human Epigenome Consortium provides public access to high-resolution reference human epigenome maps via a data portal bringing together ENCODE, Roadmap Epigenomics, CEEHRC (Canadian Epigenetics, Environment and Health Research Consortium), and other data resources. It interfaces with UCSC, Ensembl and WashU browsers as well as Galaxy for data processing. http://ihec-epigenomes.org/
UCSC genome browser	This widely used browser has many tracks which are useful for annotation; multiple SNP and variant tracks as well as tracks for resources such as ENCODE-integrated regulation and GTEx gene expression. https://genome.ucsc.edu/
Ensembl genome browser	An extensive resource of publicly available downloadable data along with a genome browser containing regulatory annotations, again including multiple ENCODE data tracks. https://www.ensembl.org/index.html
WashU Epigenome Browser	A browser specifically designed for epigenetic data; the usual SNPs, variation and ENCODE data are available, as well as additional epigenomic datasets from IHEC. http://epigenomegateway.wustl.edu/
GTEx	The Genotype Tissue Expression project is a database of tissue-specific gene expression and regulation data with downloadable and browsable QTLs, levels of expression, H3K27ac ChIP-seq and DNA methylation data. https://www.gtexportal.org/home/
GEO	Gene Expression Omnibus is a public functional genomics data repository supporting Minimum Information About a Microarray Experiment (MIAME)-compliant data submissions. Array- and sequence-based data are accepted. Tools are provided to help users query and download experiments and curated gene expression profiles. https://www.ncbi.nlm.nih.gov/geo/
METABRIC	The Molecular Taxonomy of Breast Cancer International Consortium is a large dataset of breast tumours and matched normal tissue with clinical, gene expression, copy-number aberrations (CNA), and SNP data available via cBioPortal. https://www.cbioportal.org/study/summary?id=brca_metabric
TCGA	The Cancer Genome Atlas is a conglomeration of over 20,000 primary tumours and matched normal tissue across 33 cancer types with datasets encompassing clinical, whole exome, whole genome, DNA methylation, gene expression, microRNA and proteomic profiles. https://www.cancer.gov/tcga
ICGC	International Cancer Genome Consortium is a collection of 86 cancer genome profiling projects, including datasets generated by the TCGA consortium. These datasets include clinical, whole exome, whole genome, DNA methylation, gene expression, microRNA and proteomic profiles. https://dcc.icgc.org/
PCAWG	The Pan-Cancer Analysis of Whole Genomes from ICGC and TCGA includes more than 2600 cancer whole genomes across 38 cancer types explored for somatic and germline variation with particular emphasis on non-coding RNAs, cis-regulatory sites and large structural alterations. The data portal contains somatic and germline mutations (controlled access), DNA methylation, gene expression and clinical data. https://dcc.icgc.org/pcawg
CCLE	The Cancer Cell Line Encyclopedia is a data portal including 1457 cancer cell lines encompassing gene and protein expression, DNA methylation, miRNA, mutation and CNA data. https://portals.broadinstitute.org/ccle

In this review, we focus first on methods for selecting a subset of CCVs for which there is evidence of functionality; we have summarised high-throughput methods for identifying candidate-regulatory sequences (CRS) and then testing them using a functional output. We next describe techniques for prioritising putative target genes and finally methods for linking CCVs to those target genes. Where possible, we have reviewed these tools specifically in the context of breast cancer GWAS; where these tools have not yet been applied to breast cancer GWAS, we have illustrated their potential in the context of other disease outcomes or non-disease phenotypes.

## Defining candidate-regulatory sequences (CRS)

**Fig. 1:**
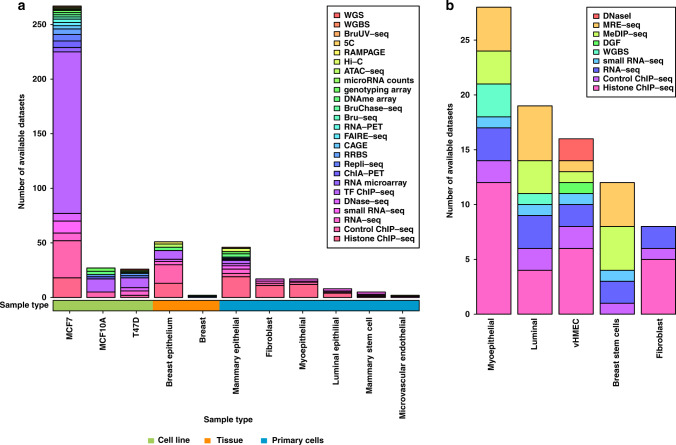
Summary of data generated in breast-relevant cell lines, tissue and primary cells that are available through ENCODE and Roadmap Epigenomics. Datasets that are available through (**a**) ENCODE and (**b**) Roadmap Epigenomics are summarised as bar plots. Different data types are colour-coded as indicated in the keys. The cell or tissue types in which the data were generated are shown on the *x* axis with the number of datasets available in each of these cell or tissue types on the *y* axis.

On the assumption that both the promoters (Table 1) and the more distal elements that regulate target genes (in particular enhancers, Table 1) will be active in one or more of the cell types that comprise breast tissue, a first step in the process of prioritising CCVs for follow-up studies is aligning the CCVs with markers of open chromatin (DNase-seq, ATAC-seq and FAIRE-seq, Table 1), active histone modifications (particularly H3K27ac, H3K4me1 and H3K4me3, Table 1) and transcription-factor- (TF, Table 1) binding sites generated in these cell types (Table 3). ENCODE, with the aim of building a comprehensive list of “regulatory elements that control cells and circumstances in which a gene is active” (https://www.encodeproject.org/), provides a rich source of these data for download and/or browsing through web portals such as the WashU Epigenome Browser (https://epigenomegateway.wustl.edu/; Table 2), the UCSC genome browser (https://genome.ucsc.edu/ Table 2) or Ensemble (https://www.ensembl.org/ Table 2). In addition to providing these datasets individually, ENCODE has generated a Chromatin State Segmentation by Hidden Markov Model (ChromHMM) function, which integrates ChIP-seq data for eight histone modifications and CTCF (Table 1) binding, to predict 15 chromatin states. Data generated in breast-relevant cell lines, tissue and primary cells are summarised in Fig. 1a and Supplementary Table 1. The majority of datasets and data types have been generated in MCF-7 cells (*N* = 267 out of a total 468 datasets), the most widely used cell-line model for oestrogen-receptor-positive breast cancer. In particular, there are ChIP-seq data for 117 TFs, including the three TFs that “define” the ER+ transcriptome (ESR1, FOXA1 and GATA3, Table 1) [32–35] generated, for example, in the presence and absence of oestradiol. By contrast, the Roadmap Epigenomics project [36] uses primary ex vivo tissues to generate normal epigenomes, these are arguably more relevant for analyses of breast cancer risk (see the eQTL section below), but the range of data types is, inevitably, more limited (Fig. 1b and Supplementary Table 1). While ENCODE and Roadmap Epigenomics are arguably the most comprehensive and widely used resources, other consortia-based resources using standardised sample preparation and assay protocols exist (summarised in ref. [37] and Table 2) and, now that data deposition is often a condition for publication, resources such as Gene Expression Omnibus (GEO, https://www.ncbi.nlm.nih.gov/geo/; Table 2) provide access to many additional (non-standardised) datasets.

**Table 3:** Methods for identifying putative target genes and functional variants.

Method: summary	Advantages	Disadvantages
*Defining candidate-regulatory sequences (CRS)*		
In silico alignment: Alignment of “local” genes and credible variants with markers of open chromatin, active histone marks and/or transcription factors. Reviewed in Klein and Hainer [103].	High-throughput in silico analysis Multiple data sources, widely available through, for example ENCODE and Roadmap Epigenomics Project (box 2). Primary cell data available through Roadmap Epigenomics Project. Can be combined into an algorithm.	The relevant tissue and/or cell type is not necessarily known. Biased towards cell lines (MCF-7, MCF 10A and T-47D) and tissue (breast epithelium) rather than primary cells (Fig. 1a) Limited markers/TF in primary cells (Fig. 1b) By combining data sources, algorithms lose granularity; can use a weighting scheme for different data types but these by definition require a series of assumptions about the hierarchy of data sources.
*Functional outputs for CRS*		
MPRA: Massively Parallel Reporter Assay [45, 46], plasmid-based high-throughput approach to reporter gene assays. CRS are placed upstream of a reporter gene driven by a minimal promoter and barcodes are inserted in the 3’UTR of the reporter gene. The activity of the CRS is measured by pairing its RNA expression to the transcribed barcodes.	High-throughput functional readout of CRS and variants within those sequences across the whole genome.	Limited to cells that can be easily transfected. The length of the sequences tested is restricted by the length of oligos that can be synthesised (~200 bp). Episomal assay. May be confounded by possible effects from promoter-binding proteins.
lenti-MPRA [50]: modification of MPRA that uses lentiviral vectors as opposed to plasmids.	Broadens the range of cells and tissue types that can be used, to include hard-to-transfect cell types. Barcodes cloned into the 5’ UTR to reduce the distance between the CRS and barcode and hence, the risk of CRS-barcode swapping. Integration of viral vector provides “in-genome” readout. Using on average >50 barcodes per CRS reduces the impact of binding of RNA-associated factors and RNA stability on the results.	The length of the sequences tested is restricted by the length of oligos that can be synthesised (~200 bp). May be confounded by possible effects from promoter-binding proteins.
STARR-seq [47]: Self-Transcribing Active Regulatory Region sequencing, plasmid-based high-throughput reporter gene assay in which the CRS itself is used as the barcode. CRS are cloned downstream of the reporter gene in the 3’UTR. The activity of the CRS is measured by comparing the amount of RNA produced relative to the amount of genomic DNA in the STARR-seq library.	The elimination of barcodes simplifies the library and allows screening of complex libraries. CRS are cloned rather than synthesised; the length of CRS are limited only by cloning efficiency and a range of 150–1500 bp is possible.	Enhancer activity may be confounded by effects from the binding of RNA-associated factors and the stability of the assayed RNA sequence. Episomal assay. Limited applicability to mammalian genomes due to their size and complexity; has been applied to human cells using selected bacterial artificial chromosomes.
CapStarr-seq [31]: modification of STARR-seq which incorporates a sequence capture step.	Overcomes limited applicability to mammalian genomes by incorporating a sequence capture step to focus on regions of interest.	Enhancer activity may be confounded by effects from the binding of RNA-associated factors and the stability of the assayed RNA sequence. Episomal assay.
GRO-seq [48]: Global nuclear Run-On sequencing, captures nascent and newly synthesised RNA, by bromodeoxyuridine (BrUTP) labelling of transcripts followed by immunoprecipitation of labelled transcripts with an antibody against BrUTP.	Assesses transcriptional regulation and activity across the whole genome. Sensitive, with a resolution of 10 bp. Robust nascent transcriptome profiles, including short-lived enhancer RNAs Capable of assessing RNAPI, RNAPII, and RNAPIII dynamics and processing properties. Generates precise quantification of promoter-proximal RNA polymerases. Low contamination of processed RNA.	Laborious assay. Requires a high input of cells (~1 × 10^7^). In vitro assay. Regulatory factors bounded to the polymerase might be eliminated by the use of sarkosyl to prevent de novo initiation of transcription.
fastGRO-seq [56]: modification of GRO-seq using 4-thio ribonucleotide (4-S-UTP) labelling followed by biotin tagging of the 4-S-UTP residues which are then captured using streptavidin beads.	More efficient assay time wise and in terms of cell input (0.5 × 10^6^) cells required. Can be used to analyse tissue and primary cells. Highly reproducible. Low contamination of processed RNA.	In vitro assay
PRO-seq [54]: Precision nuclear Run-On sequencing, modified GRO-seq assay that incorporates biotinylated nucleotides into the 3′ end of the nascent RNA and uses biotin–streptavidin pulldown.	High resolution (single nucleotide) Low contamination of processed RNA.	Laborious assay. Requires a high input of cells (~1 × 10^7^). In vitro assay. The RNA polymerase position at the beginning of transcription is mostly lost and so, it may not generate a precise quantification of promoter-proximal RNA polymerases.
TT_chem_-seq [57]: Transient Transcriptome chemical sequencing. Captures nascent and newly synthesised RNA using 4-thiouridine (4SU) labelling, uses hydrolysis instead of sonication to fragment RNA, biotin tagging of the 4SU residues and biotin streptavidin pulldown.	In vivo assay, based on metabolic labelling of RNA which minimises any variability or cellular stress. 4SU labelling is relatively easy to perform and control which is important when handling multiple samples. Highly reproducible.	Identification of regions of active transcription is limited to a resolution of 20–500 nucleotides which is the RNA fragment size range obtained after fragmentation. High contamination of processed RNA
*Identifying putative target genes*		
eQTL [59, 60]: Expression of Quantitative Trait Locus analysis: Test of association between gene expression (measured by RNA-seq now, previously microarray) and genotype.	Direct test of genotype–phenotype association. Can test local (generally defined as ≤1 to 2 Mb) and distant (>1 to 2 Mb) genes.	The relevant tissue and/or cell type is not necessarily known Limited availability of appropriate tissue and/or primary cell data, particularly large series of “normal” tissue/cells Steady state mRNA levels may not be relevant phenotype.
Colocalization [66]: Extension to individual SNP:eQTL approaches. Uses multiple variants and compares the distribution of summary statistics from eQTL and GWAS.	Reduces false positives by comparing distributions of summary statistics (as opposed to individual variants). By using gene expression data from multiple tissues, can be informative regarding “causal tissues”.	Limited availability of appropriate tissue and/or primary cell data, particularly large series of “normal” tissue/cells Steady state mRNA levels may not be relevant phenotype.
LDSC-SEG [76], DESE [77], CoCoNet [78]: Examples of **s**tatistical methods that use gene expression and GWAS data to infer causal tissues. These, and additional such methods, are reviewed in (reference [79]).	Requires gene expression but not eQTL data (i.e., does not require genotypes to be associated with the gene expression). Can help to inform relevant tissue or cell type for in vitro experiments.	Assumes that driver genes will be relatively highly expressed in the most disease-relevant tissue types LDSC-SEG additionally assumes that SNPs near such driver genes will be enriched for heritability Limited by the availability of gene expression data in relevant tissues or cell types Steady-state mRNA levels may not be relevant phenotype.
Transcriptome-wide association studies (TWAS [68, 69])**:** eQTL cohorts are used to develop models of expression variation on a per gene basis; models are then used to predict gene expression for individuals in GWAS and test for association between gene expression and outcome.	Informative both for discovery (new risk loci) and for inferring target genes at “known” GWAS loci. Can help to inform relevant tissue or cell type for in vitro experiments.	Limited availability of appropriate tissue and/or primary cell data, particularly large series of “normal” tissue/cells Steady state mRNA levels may not be relevant phenotype.
Comparison with somatically mutated cancer genes (boxes 1 and 2): in silico analysis of somatic variation in tumours using whole genome or exome sequences.	Provides robust evidence for a functional role in cancer either on an ad hoc basis or by comprehensively comparing genes that are local (generally within 1 Mb of a locus) with lists of somatically mutated genes.	Undermines the “discovery” aspect of GWAS; only provides confirmation that the concept of an unbiased GWAS approach is sound.
*Linking CRS with putative target genes*		
CHi-C [96, 97]: Capture Hi-C. Chromatin-interaction method that exploits the 3D proximity of long-range regulatory elements and the genes that they regulate using formaldehyde cross-linking of chromatin followed by sequence capture to focus on regions or features of interest.	High throughput Potentially two-sided (i.e., either GWAS loci or the promoters of putative target genes can be used as “baits”). Agnostic	CHi-C interaction peaks will include interactions that are structural (e.g. driven by CTCF and/or cohesion) rather than regulatory in situ CHi-C requires large numbers of cells (new Hi-C kits are reducing the numbers of cells required). Most data have been generated in cell lines, not primary cells—in part due to the requirement for large numbers of cells Interaction peaks are defined by a viewpoint—i.e., linkage-disequilibrium blocks or promoters.
ChIA-PET [98]: Chromatin Interaction Analysis by Paired-End Tag sequencing, HiChIP [10]: combination of 3C or Hi-C technology with chromatin immunoprecipitation.	High-throughput two-sided, but only when both ends of the interaction are captured (i.e., they both involve the TF or histone modification of choice).	ChIA-PET requires large numbers of cells; HiChIP less so, particularly with new HiChIP kits Very little published data – ChIA-PET data generated in MCF-7 for ESR1, MCF-7, and POLR2A as part of ENCODE. Interaction peaks are defined by a viewpoint—the TF or histone modification used for the immunoprecipitation.
CRISPR-Cas9**:** Genome editing system in which a guide RNA delivers a Cas9 nuclease to a specific DNA locus where the nuclease makes a double-stranded break. Genetic changes are introduced during the DNA repair process. These genetic changes could be a specific nucleotide change (knock-in using homologous directed repair (HDR)), a DNA sequence or an entire gene could be removed (knock out).	In genome (as opposed to episomal) assay Genome can be precisely manipulated by the CRISPR system’s ability to introduce specific changes. Relatively simple assay to design and perform.	Random modifications can occur in off-target sequences. It is not suitable for all cells; some do not use homologous directed recombination as their main repair pathway, some cells are non-diploid due to genome instability. HDR efficiency is relatively low; for GWAS CCVs where a single base change is often required, base editing approaches may provide an alternative (reviewed in ref. [104]).
CRISPRi (CRISPR interference), CRISPRa (CRISPR activation [105]) and other CRISPR modifications**:** techniques use a deactivated Cas9 (dCas9) fused to an effector domain eg Kruppel associated box (KRAB) which spreads repressive histone modifications (CRISPRi) or an activator eg VP64-p65-Rta (VPR, CRISPRa). Reviewed in ref. [104], with recent additions including CRISPR knock-in [106] and repression CRISPRoff [107].	Highly specific assays, multiple target genes can be modulated simultaneously and the introduced genomic changes are potentially reversible.	Can be challenging to design sgRNA proximal to the region of interest. It is important to design multiple sgRNA for each target as they have variable efficiency.

These data are valuable resources that have already been widely used to define CRS and prioritise CCVs for follow-up studies (see locus-specific annotation studies below). Given that risk reflects early events that precede the somatic genome, the predominance of data generated in a breast cancer cell line (MCF-7) is potentially limiting progress in this area; in this context, Fachal et al. reported that 73% of active enhancer regions (as defined by ENCODE ChromHMM) that overlapped ER+ CCVs in MCF-7 cells were not active in normal human mammary epithelial cells [5]. Two additional rich resources have recently been reported; using genomic DNase-I footprinting integrated with TF-recognition sequences, condensed onto a common sequence axis for closely related TF family members, Vierstra et al. have generated a high-resolution genome-wide consensus TF-footprint index in 243 human cell and tissue types, including a breast cancer cell line (T-47D), a normal mammary epithelial cell line (MCF10a) and normal mammary fibroblasts [38]. Contemporaneously, Domcke et al. developed single-cell ATAC-seq (sci-ATAC-seq3), which they combined with single-cell gene expression data across a broad range of human foetal tissues, to create an atlas of linked cell-type-specific enhancers and genes that have the potential to inform our understanding of cell-fate specification and maintenance in normal tissue [39]. These data, which can be accessed through the ENCODE portal and the Descartes website, respectively (Table 2), have the potential to transform our ability to define CRS and evaluate CCVs.

## Functional outputs for validating candidate-regulatory sequences

Markers of open chromatin, active histone modifications, TF binding and/or chromatin-interaction peaks (Table 3) have been used to map millions of sites with regulatory potential across the genome [31, 40]. The proportion of these predicted elements that truly function as, for example, enhancer elements, is not known, but estimates range from 12% to up to 90% [41–44]; clearly, functional validation is required. High-throughput methods for functional validation can assay expression of experimental constructs (massively parallel reporter gene assays (MPRA [45, 46]), self-transcribing active regulatory region sequencing (STARR-Seq [47])) or expression of nascent transcripts (eRNAs) from the predicted enhancer element in a “normal” genomic context (GRO-Seq [48], Table 3).

The classic method for assaying enhancer activity is the reporter gene assay [45]. Briefly, a candidate- regulatory sequence (CRS) is cloned into a reporter construct comprising a minimal promoter and a reporter gene with a quantifiable output such as green fluorescent protein (GFP), β-galactosidase (LacZ) or luciferase. In the context of breast cancer GWAS annotation, the reporter construct(s) are then transfected into a breast cancer cell line and reporter activity is assayed to determine whether the CRS enhances transcription and whether this activity is allele-specific. MPRA (46) and STARR-seq [47] were both developed to “high-throughput” reporter gene assays allowing several thousand putative CRS to be tested for enhancer activity simultaneously (Table 3). Both methods use RNA expression driven by the CRS either by pairing it to a transcribed barcode in the 3’ or 5’ UTR (Table 1) of the reporter gene (MPRA), or by using the CRS itself as a barcode (STARR-seq, CapStarr-seq [49]). Lenti-MPRA [50] (Table 3), a lentivirus-based version of MPRA, extends this technology to cell types that are “hard-to-transfect” and as lentiviruses integrate into the genome, produces “in-genome” readouts as opposed to episomal readouts (Table 1). To our knowledge, these high-throughput versions have not yet been used in the context of breast cancer GWAS, but the potential of lenti-MPRA to recapitulate an exquisitely regulated programme of temporal and cell-type-specific gene expression was demonstrated recently using neural induction from human pluripotent stem cells (hPSCs, Table 1) as a paradigm [43].

Non-coding transcription (eRNA, Table 1) is a defining feature of active enhancers [51]; these nascent RNAs can be assayed using high-throughput adaptations of a technique (nuclear run-on assays) that was originally developed to measure rates of transcription [52, 53]. GRO-seq [48], one of these high- throughput adaptations (Table 3), rather than incorporating radionucleotides (as used in the nuclear run- on assays) uses bromodeoxyuridine labelling of nascent RNA transcripts followed by immunoprecipitation using an antibody against bromodeoxyuridine. Subsequent methods (PRO-seq [54], mNET-seq [55], fastGRO-seq [56] and TTchem-seq [57], Table 3) have introduced modifications to this protocol that involve 4-thiouridine labelling, incorporating a biotin tag and/or hydrolysis rather than sonication to fragment the nascent RNAs (Table 3). In the context of breast cancer specifically, Franco et al. generated GRO-seq data in a series of 13 breast cell lines (11 cancer and two immortalised “normal” breast cell lines), and combined these with RNA-seq and ChIP-seq data to investigate whether subtype-specific gene expression programmes control breast cancer pathogenesis [58].

Reporter gene assays have been used to differentiate functional variants from correlated variants in several locus-specific studies (see locus-specific annotation studies below). Lenti-MPRA has several advantages that are likely to render these individual assays obsolete, specifically, by generating high- throughput data that capture the “in genome” activity of several thousand CCVs simultaneously in “hard-to-transfect” primary cells. Comparing GRO-seq with other enhancer marks (open chromatin and active histone modifications), Franco et al. demonstrated that GRO-seq identifies smaller numbers of high- specificity enhancers [58] and recent adaptations to the protocol reduce cell numbers, such that it should be possible to generate these data too, in primary cells [56]. However, without a formal comparison of these data types in the same cell types, and an understanding of ground truth (presumably in the form of extensive well-characterised positive and negative controls), it is not possible to say which methodology performs best in terms of providing a functional readout for bona fide regulatory elements.

## Identifying putative target genes

The logical first step to identifying putative target genes is expression of quantitative trait locus (eQTL) analysis (Table 1), i.e., to test for association between genotype of a GWAS-risk SNP (or a correlated variant) and gene expression (generally steady-state levels of mRNA). It has been shown previously that levels of gene expression are genetically determined (reviewed in ref. [59]) and therefore steady-state levels of mRNA can be considered as an intermediate phenotype (Table 1) that potentially mediates a causal association between a regulatory variant and a complex disease such as breast cancer. As such, a statistically significant eQTL with a gene that maps locally to a GWAS signal (generally defined as within 1Mb) in breast tissue provides strong evidence of a causal association between this “target gene” and breast cancer risk. In their integrative eQTL-based analysis of 15 published breast cancer risk loci, Li et al. identified three cis-associations (2q35-*IGFBP5*, 5q11-*SETD9* and 16q12-*TOX3,* Table 1) and three trans-associations, which they defined as associations with downstream genes for which there was evidence of regulation by a TF mapping locally to the GWAS locus [60] (6q25-*ESR1*, 9q31-*KLF4* and 8q24-*MYC*).

Normal breast tissue and breast tumours have both been used as sources of gene expression data for eQTL analyses [2, 5, 60–62]; while normal tissue is arguably more relevant for analyses of breast cancer risk (reflecting early events that precede the somatic genome), there is greater availability of expression data from breast tumours. Publicly funded datasets that are available to researchers include the Genotype-Tissue Expression project (GTEx [63], https://www.gtexportal.org/home/), The Cancer Genome Atlas (TCGA, https://www.cancer.gov/about-nci/organization/ccg/research/structural-genomics/tcga) METABRIC [64] and the Pan-Cancer Analysis of Whole Genomes (PCAWG [65], https://dcc.icgc.org/pcawg; Table 2). In addition to the germline variation (Table 1) that is being investigated by GWAS, gene expression in the somatic genome (Table 1) can be influenced by copy number (Table 1) and epigenetic changes such as promoter hypermethylation (Table 1); however, methods that adjust for these somatic events have been developed [59, 60], and breast tumours have been widely used in eQTL analyses to identify “target genes” of breast cancer GWAS-risk loci [2, 5, 60–62].

Colocalisation analysis provides an extension to individual SNP:eQTL lookup approaches, by using multiple variants and comparing the distribution of summary statistics from both eQTL and GWAS- association signals, colocalisation reduces false-positive associations and provides a greater degree of confidence that an association between a locus, gene expression and disease outcome is causal [66]. Using eQTL data generated in normal breast tissue from 396 individuals (GTEx v.8) and GWAS summary data, Beesley et al. [67] carried out a colocalisation analysis of the BCAC fine-scale mapping breast cancer risk regions [5]. They identified 17 genes at 14 loci at which the GTEx eQTL associations were statistically significant (defined as *P* < 10^−6^). For 11 of these genes, the eQTL SNPs colocalised with strong GWAS signals (*P* < 10^−6^, based on multinomial logistic-regression analysis) supporting a causal association. However, the extent to which these associations were replicated in TCGA data was limited and levels of orthogonal support varied [67].

Transcriptome-wide association studies (TWAS) further extend the concept of gene expression levels as an intermediate phenotype for both identifying target genes at known GWAS loci and the discovery of novel risk loci [68, 69]. Briefly, eQTL cohorts with gene expression and genotype data are used to develop models of expression variation on a per-gene (as opposed to per-SNP) basis. These models are subsequently used to predict levels of gene expression for individuals in a GWAS cohort, and test for the association between predicted levels of expression and, for example, breast cancer risk. TWAS methodology and tools for implementing this methodology have been published [68, 69] and were reviewed recently [70]. In the context of this current review specifically, breast cancer TWAS using breast tissue, whole blood, adipose tissue and immune cells as the gene expression cohorts has been reported [71–75], all report genes for which genetically regulated expression levels may be associated with breast cancer risk at both novel loci and known GWAS loci. While these analyses have exclusively used breast tissue and/or cell types that would be present in breast stroma (i.e., immune cells and adipocytes), Michaildou et al. [2] carried out a cell-type-specific enrichment analysis of genome-wide SNP heritability and found significant enrichment of active histone modifications in several non-breast- tissue types, including stomach, rectal and colonic mucosa.

Statistical methods that use gene expression and GWAS data to infer “causal tissues”, including colocalisation analysis [66], linkage-disequilibrium score regression applied to specifically expressed genes (LDSC-SEG [76]), driver-tissue estimation by selective expression (DESE [77]) and Composite likelihood-based Covariance regression Network model, (CoCoNet [78]) have been developed. For a comprehensive comparison of statistical approaches for integrating genome-wide datasets for the functional annotation of GWAS loci, the reader is referred to a recent review by Cano-Gamez and Trynka [79]. Briefly, these methods use gene expression data in multiple tissue types to determine whether disease heritability is directly associated with tissue-specific gene expression patterns (DESE [77]), enriched in regions surrounding genes that show high levels of tissue-specific expression (LDSC-SEG [76]) or co-expression in specific tissue types (CoCoNet [78]). Predicated on the assumption that driver genes will be “relatively” highly expressed in the most disease or trait-relevant tissue types, these methods integrate tissue-specific gene expression data with disease or trait-specific GWAS data to infer causal tissues and driver genes.

Breast tissue is heterogeneous; the parenchyma comprises a branched structure of ducts and lobules composed of specialised epithelial cells (an inner layer of luminal cells and an outer layer of myoepithelial cells) surrounded by stroma, connective tissue populated by fibroblasts, myofibroblasts, endothelial cells, adipocytes and immune cells [80]. Given that gene expression is cell-type-specific, not “tissue-specific”, several of the large data series have used microdissection to select out regions of the tumour that predominantly comprise cells of epithelial origin. This approach, however, assumes that the target gene(s) act in a cell-autonomous (Table 1) manner. Alternative in silico approaches to deconvolute cell-type-specific expression profiles have also been developed [81–83]. Whilst these have mainly been used to test for the association between clinical covariates and breast cancer prognosis [83, 84], Seo et al. used a deconvolution approach to examine gene expression in normal breast tissue [61]. Specifically, they modelled breast tissue as comprising four different cell types (adipocytes, epithelial, inflammatory and stromal), and identified eQTL associations at published breast cancer GWAS loci in two of these cell types—epithelial and stromal cells [61]. Notably, in their recent fine-mapping analysis of 150 breast cancer risk regions, Fachal et al. reported eQTL associations in normal breast tissue (NHS [85] or METABRIC [64]) at 72 of their fine-mapping regions, several of these stand out as associations with genes that are expressed in fibroblasts [86] or immune cells [87], including *FBLN5* (fibroblasts), *MEFV* (monocytes and neutrophils) and *APOC1* (macrophages) [5].

Exome (Table 1) and, more recently, whole-genome sequencing of a large series of matched cancer genes (Table 1) has been conducted for many different site-specific cancers, including breast cancer [65, 88–90] (Table 2). Several such genes map to published breast cancer risk loci, including 10q26-*FGFR2*, 6q25-*ESR1* and 5q11-*MAP3K1* and are a priori strong candidates for playing a functional role in the association between a GWAS locus and breast cancer risk [65, 88–91]. Accordingly, several large-scale annotation analyses have prioritised lists of putative target genes by comparing them with lists of somatically mutated cancer genes, both on an ad hoc basis [62] and more comprehensively [2, 5, 92, 93]. While finding agreement between somatically mutated cancer genes and putative target genes at GWAS-risk loci provides reassuring evidence that GWAS “work”, the strength of an unbiased GWAS approach is the potential for discovering novel cancer genes, and as such, it is arguable that the more interesting target genes are those that have not already been shown to be somatically mutated cancer genes.

## Linking candidate-regulatory sequences with putative target genes

While the identification of a statistically significant eQTL between a GWAS SNP (or correlated variant) and a gene that maps locally to a GWAS signal provides strong evidence of a causal association, the absence of an eQTL does not preclude a gene from a functional association. Steady-state levels of mRNA will not capture expression during a particular developmental window, in response to an environmental stimulus or in a specific cell type that occurs at a relatively low frequency within the breast [59]. In addition, eQTL analyses alone cannot distinguish between functional variants and correlated variants.

In the first generation of GWAS, a “nearest gene”, a “nearest expressed gene” or even a “nearest plausible gene” approach was often used to infer the target gene(s) and define the locus. For example, the 10q26 breast cancer risk locus was referred to as the *FGFR2* locus before Meyer et al. carried out functional studies that implicated regulation of *FGFR2* expression through allele-specific binding of E2F1 and FOXA1 as the likely mechanism by which this locus influences risk [21, 94]. Linking potentially functional variants and/or the CRS to which they map, with the genes they regulate, requires consideration of the 3D genome [95]. Physical interactions between cis-acting regulatory elements and transcriptional start sites (TSS, Table 1) can occur over linear distances of ≥1 megabase (Mb), can skip over multiple intervening genes and are not exclusive; on average, each promoter interacts with 3.9 distal regulatory elements and each distal regulatory element interacts with 2.5 promoters [31]. The chromosome-conformation capture (3C, Table 1) family of methods is used to identify long-range interactions based on (3D) chromatin conformation in the cell. Briefly, spatially proximal segments of DNA are covalently linked using formaldehyde cross-linking of chromatin in intact nuclei, this is followed by restriction-enzyme fragmentation, ligation of linked DNA fragments and finally detection and quantification of ligation products. In the original 3C protocol, ligation products were identified one at a time using polymerase chain reaction (PCR) with locus-specific primers (a “one-by-one” approach); by contrast, Hi-C (Table 1) is the “all-by-all” method used to identify chromatin interactions genome-wide [95]. To generate the high-resolution data required for cataloguing interaction peaks at kilobase (or less) resolution, targeted chromatin-interaction methods focussed on GWAS linkage-disequilibrium (Table 1) blocks [62, 96] or annotated promoters [97] have been used (Table 3). We developed region-capture Hi-C (rCHi-C) specifically to identify target genes at three breast cancer-associated gene deserts [96]; we and others have expanded this approach to identify putative target genes at up to 139 independent breast cancer signals [62, 92]. Chromatin Interaction Analysis by Paired-End Tag Sequencing (ChIA-PET [98]) and HiChIP [10] are chromatin-interaction methods that combine 3C (ChIA-PET, Table 3) or Hi-C (HiChIP, Table 3) with an immunoprecipitation step targeting, for example, the histone modification H3K27ac. To our knowledge, there have been no ChIA-PET or HiChIP studies carried out in breast cancer or “normal” mammary epithelial cells. Chandra et al., however, demonstrated the potential of HiChIP to define functional eQTL associations; combining HiChIP for the histone modification H3K27ac in different types of primary immune cells with eQTL datasets from matched cell types, they identified a subset of “promoter interacting eQTLs” that were associated with cell-type-specific expression of target genes [10].

However, it is arguable that, based on the assays described above, the evidence that associations between CRS (harbouring one or more CCVs) and target gene expression are causal is at best circumstantial; direct evidence would require perturbation of the CRS, resulting in an alteration to levels of expression of the target gene. This type of direct evidence is achievable using CRISPR genome editing (Table 1). In a follow-up analysis of the 11q13 breast cancer risk locus, Betts et al*.* used CRISPR interference (CRISPRi) to introduce repressive histone modifications at an enhancer element (annotated by the most significant GWAS SNP at this locus) and demonstrated that this resulted in reduced levels of expression of two long noncoding RNAs (*CUPID1* and *2*) and the presumed target gene *CCND1* [22]. We have recently shown that targeting a catalytically inactive Cas9 fused to an activating VPR domain (CRISPRa) to an enhancer element at the 2q35 breast cancer risk locus increases expression of *IGFBP5* (mapping ~400 kb distal) but neither of the neighbouring genes *IGFBP2* and *RPL37A* (~460 kb and ~600 kb, respectively) [99]. A genome-wide framework for mapping gene regulation using CRISPRi has been developed; in this approach, using a high multiplicity of infection, random combinations of CRS were perturbed in the erythroleukaemia cell line K562 and expression of target genes (defined as K562-expressed genes within 1Mb of the CRS) was assayed using single-cell RNA-seq [100]. To our knowledge, this type of genome-wide approach has not yet been used in the context of breast cancer GWAS loci.

Demonstrating an association between genotype of a GWAS-risk SNP (or correlated variant) and gene expression arguably still provides the most direct evidence that a gene plays a causal role in influencing disease risk. Statistical methods that consider multiple variants and compare the distribution of summary statistics (rather than individual eQTL:SNP lookups) provide more robust evidence and may contribute to our ability to infer causal tissues. However, these methods, which rely on steady-state levels of mRNA, will not capture expression during a particular developmental window, in response to an environmental stimulus or in a specific cell type. As the costs of single-cell RNA-seq continue to decrease, this may in part be addressed by increasing availability of large single-cell RNA-seq and genotype datasets for future eQTL-type analyses. In our view, chromatin-interaction methods and CRISPR perturbation can still add to, or detract from, the weight of evidence for a given variant influencing a particular “target gene”. There are advantages to CHi-C as a chromatin-interaction method; CHi-C makes no assumptions about the nature of the regulatory interaction and new kit-based methods (https://arimagenomics.com/, https://dovetailgenomics.com/) have the potential to improve resolution and reduce input in terms of numbers of cells. Ultimately identifying target genes and causal variants, robustly, is likely to require multiple data types; the most informative approaches will inevitably vary from locus to locus and depend on the mechanism that links variant, gene and disease risk.

## Locus-specific functional annotation studies

Locus-specific functional annotation studies for at least 17 loci (defined for these purposes as chromosomal regions) have been reported by BCAC investigators and collaborators at 1p11.2 [25], 2q33 [27], 2q35 [12, 13, 99], 4q24 [14], 5p15.33 [28], 5p12 [24], 5q11.2 [15], 6q25 [16], 8q24 [17], 9q31.2 [18], 10q21.1 [19], 10q26 [21], 11q13 [23], 12p11 [26], 12q24 [92], 17q22 [20] and 19p13 [29]. These analyses, published predominantly prior to the recent global fine-mapping analysis, begin with locus-specific fine-scale mapping to define independent signals and CCVs. At the vast majority, this has resulted in too many signals and variants for individual functional assays without first prioritising a subset of CCVs by aligning them with regions of open chromatin, active histone modifications and/or TF-binding sites. Similarly, potential target genes (frequently defined as genes that map within 1 or 2Mb of the most significant SNP) tend to be selected on the basis of eQTL analyses and genome-wide chromatin- interaction data (ChIA-PET and/or Hi-C). On this basis alone, some studies have proposed possible target gene(s) and provided lists of variants that warrant further investigation [14, 17, 20, 25, 26]. Other studies have followed up a subset of variants and genes using functional assays and, in some instances, report more robust evidence for a causal variant (or variants), a target gene (or genes) and a mechanism by which the causal variant influences the expression of the target gene to impact breast cancer risk. Target genes include well-documented breast cancer genes (*MAP3K1* at 5q11.2 [15], *ESR1* at 6q25 [16], *FGFR2* at 10q26 [21] and *CCND1* at 11q13 [23]), TFs (*KLF4* at 9q31.2 [18], *NRBF2* at 10q21.2 [19] and *TBX3* at 12q24 [92]), a putative tumour suppressor gene (*IGFBP5* at 2q35 [12, 13, 99]), a methylcytosine dioxygenase (*TET2* at 4q24 [14]) and a ribonucleoprotein polymerase that maintains telomere ends (TERT at 5p12 [24]). The majority of studies propose a mechanism in which allele-specific binding of a TF (or TFs) influences the expression of the target gene; most commonly, it is the allele-specific binding of one of the three factors that define the ER+ transcriptome (ESR1, FOXA1 and GATA3) [33–35] that is implicated. There is however an element of self-fulfilling prophecy to this: ESR1, FOXA1 and GATA3 ChIP-seq data in breast-relevant cell types are widely available and inevitably incorporated into the process for prioritising variants for follow-up studies.

While some of these locus-specific studies have provided insight into the mechanisms that influence risk at individual loci, it is clear, given the size of the task, that high-throughput approaches are required. In the global fine-scale mapping analysis recently published by the BCAC, Fachal et al. used two approaches to incorporate genome-wide functional data into their analyses [5]; they used a Bayesian approach (PAINTOR [101]) that combines genetic association, linkage disequilibrium and enriched genomic features to determine variants with high posterior probabilities of being causal (PPs) and then analysed both of these, and the CCVs from their fine-scale mapping by multinomial logistic regression, using their integrated-expression quantitative trait and in silico prediction of GWAS targets (INQUISIT). Inevitably, the range of assays and cell types used to generate the genomic features that are incorporated into PAINTOR, and those upon which INQUISIT predicts target genes, is limited by the available data: of the 811 genomic features incorporated into INQUISIT, 362 (44.6%) were generated in the oestrogen-receptor-positive breast cancer cell line MCF-7, and 191 (23.5%) were histone- modification ChIP-seq data. Overall, they reported 34 signals at 25 regions where there was either a single CCV or a variant for which the posterior probability was >80% (i.e., individual variants with a high a priori probability of being functional) and 191 high-confidence (level-1) target genes mapping to 88 regions. However, there remain multiple statistically indistinguishable CCVs at the majority of signals, multiple regions without high-confidence target genes and the high-confidence genes that have been predicted require validation and further (mechanistic) investigation.

## Perspective

Over the last 15 years, GWAS has transformed our understanding of the genetic architecture of common diseases such as breast cancer. To date, however, the findings of breast cancer GWAS have not led to transformative insights into disease mechanism or new approaches to disease prevention and treatment. The recently published fine-scale mapping and functional annotation that was carried out by the BCAC constitutes a major step forward, but also highlights the challenges [5]; with 152 regions, 352 independent signals and 13,367 CCVs to characterise, there is a clear need for broad-scope systematic approaches integrating statistical and functional data. It is also clear, however, that based on the functional data that are currently available, the results of this type of systematic approach (exemplified by PAINTOR and INQUISIT), still fall a long way short of deciphering the mechanism by which each locus influences a woman’s risk of breast cancer. There are clearly some critical gaps in the range of genome-wide functional datasets that are available; there is an abundance of markers that correlate with enhancer marks (histone modification and TF ChiP-seq) but little or no data for the functional validation of these candidate-regulatory sequences (MPRA, eRNAs or CRISPR screens). In addition, the vast majority of data have been generated in a single oestrogen-receptor-positive breast cancer cell line—MCF-7. Regulation of gene expression can be highly specific in terms of timing (both with respect to development and/or a stimulus) and cell type; if it is arguable that normal tissue is more relevant for eQTL analyses of breast cancer risk, it must also be arguable that normal primary cells are more relevant for functional assays. Future efforts to generate breast-relevant functional data may be better focussed on normal primary cells rather than breast cancer cell lines. In addition, as the range of single-cell technologies increases, and the cost of these methods decreases, the opportunities for generating more sophisticated functional data that more accurately reflect the cellular heterogeneity within breast tissue are also opening up [102]. In conclusion, while much work has been done, there is still much to do. There are, however, grounds for optimism; combining statistical data from fine-scale mapping with functional data that are more representative of the normal “at risk” breast, generated using new technologies, should lead to a greater understanding of the mechanisms that influence an individual woman’s risk of breast cancer.

## Supplementary information

Supplementary Data 1
